# Medical Waste Incineration Fly Ash as a Mineral Filler in Dense Bituminous Course in Flexible Pavements

**DOI:** 10.3390/ma16165612

**Published:** 2023-08-13

**Authors:** Rumpa Chowdhury, Mir Tanvir Al Biruni, Antara Afia, Mehedi Hasan, Mohammed Russedul Islam, Tanvir Ahmed

**Affiliations:** 1 Department of Civil Engineering, Bangladesh University of Engineering and Technology, Dhaka 1000, Bangladesh; rumpa.ce.buet@gmail.com (R.C.); tanvirmir444@gmail.com (M.T.A.B.); afia.tajalli@gmail.com (A.A.); mehedi@itn-buet.org (M.H.); 2 Department of Civil Engineering, Military Institute of Science and Technology, Mirpur Cantonment, Dhaka 1216, Bangladesh; russed@ce.mist.ac.bd

**Keywords:** fly ash, mineral filler, sustainable pavement, Marshall properties, heavy metal leaching, environmental impact

## Abstract

Medical waste incineration fly ash (MWIFA) contains heavy metals that are toxic by nature and pose numerous health risks. The paper deals with the suitability of MWIFA as a mineral filler in the bituminous layer as an alternative to conventional stone dust (SD) through an appropriate combination of engineering and environmental assessments. Engineering parameters, such as Marshall stability, stability loss, flow, unit weight, air voids (V_a_), voids filled with asphalt (VFA), and voids in the mineral aggregate (VMA) of the asphalt mixtures, were evaluated with varying filler ratios, from 2% to 10%. All parameters for both fillers at optimum bitumen content satisfied the Marshall Mix Design criteria. The optimum bitumen contents of all filler ratios were within the standard limit recommended by the Bangladesh Roads and Highways Department. It was found that mixes prepared with MWIFA can resist moisture effects, making them durable in the monsoon. The mixes with 5.5% MWIFA as mineral filler performed the best, whereas 9% SD filler was required to achieve similar performance. The environmental test results show no environmental restriction on stabilizing the MWIFA into paving mixtures. The mobility of heavy metals (As, Pb, Cu, Cr, Ni, Cd, Hg, and Zn) from the asphalt-MWIFA mix was insignificant. The cumulative concentrations of heavy metals (Cd, Ni, Zn, Cu, and Pb) from long-term leaching tests were far below the Dutch regulatory limit (U1). MWIFA can be considered an eco-friendly and sustainable mineral filler for the dense bituminous pavement layer.

## 1. Introduction

Medical waste is a source of pollution and infection for humans and the natural environment. With the rapid growth of healthcare facilities, enormous quantities of medical waste are generated in Bangladesh, with an annual generation rate of 93,075 tons (average rate of 0.8–1.67 kg/bed/day) [1,2]. The incineration of medical waste, followed by the dumping of ash into landfills, is the most common practice for disposal [3]. The incineration process can reduce waste by 70% but generates significant residual ashes [4]. The ashes generated from medical waste incineration are enriched with heavy metals, and exposure to these can cause damage to the environment and human health [5]. Fly ash can spread to a greater distance by the wind, which helps them enter the food chain using the air, soil, and surface water as exposure pathways and cause bioaccumulation in the food chain [6]. At present, stabilization/solidification, one of the most renowned and appropriate pre-landfill waste treatment techniques, has been adopted to alleviate the leaching toxicity of fly ash and convert the heavy metals into a stabilized insoluble structure [7,8]. Medical Waste Incineration Fly Ash (MWIFA) can be successfully stabilized in construction materials using cement, ceramic tiles, and synthetic geotextile [6,9,10]. 

Some recent research has focused on using waste materials in highway construction and road layers from the top layer to the subgrade [11,12]. Studies on using waste material in pavement construction are documented in the literature [11,12,13,14,15,16,17]. Using fly ash in asphalt pavement is promising due to its positive impacts on the performances of asphalt concrete mixtures and cost and eco-friendly characteristics [18]. Fly ash not only seals the voids in the asphalt concrete mix but also provides contact points between larger aggregate particles and thus can be an ideal filler material [19]. Fly ash has been found to provide good stability in asphalt mixtures [18,20,21]. Mistry and Roy [22] found that fly ash in hot mix asphalt (HMA) provides lesser deformation with good strength properties. 

Filler is a crucial component of the asphalt paving mix, and it modulates the properties of asphalt concrete mixtures [23]. Well-packed aggregates (coarse and fine aggregates) combined with filler are the backbone of the asphalt mixture [24]. The mixture of asphalt binder and mineral filler controls the complete performance of pavement mixtures [25]. Filler controls the mechanical properties of asphalt mixtures by providing additional contact points between larger aggregates and increasing the viscosity of asphalt binders [26,27]. The Asphalt Institute recommends 4% to 8% filler usage in asphalt concrete [28].

Several researchers have investigated the suitability of using fly ash as mineral filler in bituminous mixes in recent years. Fly ash from the combustion of pulverized coal [29], thermal power plants [30,31], incinerated domestic and industrial products and wastewater sludge [32], burning coal [33], municipal solid waste incineration [34], and burning crude oil [35] have been used to evaluate and improve bituminous mixes performance in asphalt pavement. Sobolev et al. [36] studied the viability of fillers, i.e., fly ash, lime, and cement in asphalt concrete and demonstrated that adding these fillers improved the rheological properties of the asphalt. Al-Hdabi [37] found that using rice husk ash as a mineral filler improved the Marshall stability of hot asphalt mixtures more than conventional mineral fillers. Radwan et al. [38] found higher stability values and lower flow values for the coal fly ash mixes than conventional filler. The mixes showed strong moisture resistance and durability, which validated the suitability of coal fly ash filler for HMA mixes. Zhao et al. [39] showed that asphalt mixture with fly ash had more thermal susceptibility but recommended a maximum 25% fly ash ratio considering moisture intrusion.

The physical, chemical, rheological, and mechanical properties of asphalt mixtures containing fly ash from municipal solid waste used as a substitute for fine aggregate or filler material have been explored [32,39,40,41,42,43]. Owing to the attractive outcomes of these studies, the probability of using fly ash from medical waste incineration, which is highly contaminated compared to municipal solid waste in nature, as a filler material in similar applications appears to be highly promising. MWIFA as a filler material in pavement construction is comparatively recent. Only a few studies have demonstrated its feasibility as a paving material. Jaber et al. [44] studied the suitability of using residual ashes from medical waste in the base layer of pavement, and a ratio of 25% of the ashes was recommended based on Marshall properties only. According to prior experimental findings, it might be feasible for toxic MWIFA to be used as a filler material. Most studies on fly ash stabilization in asphalt pavements are confined to assessing engineering suitability without considering the environmental effects after stabilization for long-term usage. MWIFA is hazardous, and its leaching activity can cause several adverse impacts on human health and the environment [5,6]. To our knowledge, no study assessed the environmental effects of incorporating hazardous MWIFA in pavement construction. There are substantial research deficiencies in computing the environmental impacts of MWIFA-incorporated pavements, which require further investigation. Hence, a combination of appropriate environmental tests is required to evaluate the suitability of using MWIFA as filler material in bituminous mixes. 

The materials used as common fillers, such as cement, limestone, and granite powder, are not easily and economically available in countries such as Bangladesh [15]. Therefore, fly ash can be an economical alternative to more expensive filler materials. This study investigates the environmental compatibility of stabilized MWIFA as a mineral filler in the bituminous layer as an alternative to conventional fillers. The Marshall and leaching properties of asphalt paving mixes containing different proportions of MWIFA and stone dust (SD) were determined and compared with available standards and guidelines. Marshall mix designs using MWIFA and SD as fillers were performed to determine the optimum bitumen and filler contents. The heavy metal leaching characteristics of the solidified asphalt fly ash matrix were investigated to evaluate the environmental impacts. The results of this study will contribute to the developing knowledge of the engineering feasibility and environmental impacts of using fly ash from hazardous medical waste incineration in pavement construction.

## 2. Methods and Materials

### 2.1. Test Scheme

**Figure 1:**
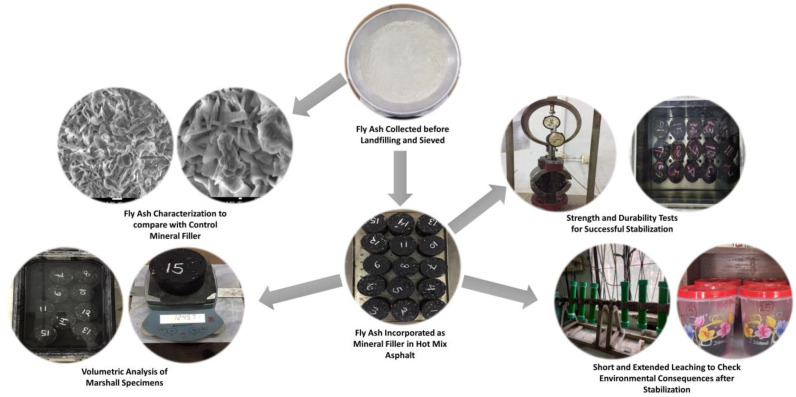
Flow chart showing the test outline.

Figure 1 presents an outline of the tests required in this research. The details will be described in the following sections.

### 2.2. Bitumen 

Considering the weather pattern and traffic volume of Bangladesh, 60/70 grade bitumen was used in this study. The properties of bitumen used in the asphalt mix with AASHTO standard designations are given in Table 1. All the properties except solubility are within the standard ranges. The lower solubility value than the standard could be due to mineral impurities in the bitumen.

### 2.3. Aggregates

**Figure 2:**
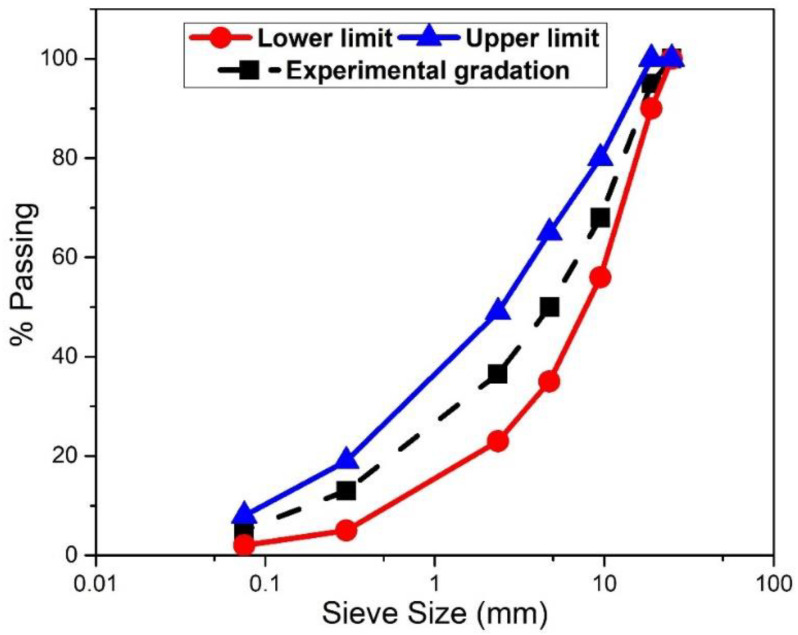
Gradation of combined aggregate.

The aggregate gradation used in this study is shown in Figure 2, which fulfills the ASTM D3515-01 [45] hot mix paving mixtures standard specification criteria for the dense mixture (mix designation D-4). An equal portion of fine aggregate was substituted by filler material while increasing the filler ratios to keep the specified total aggregate quantity constant. Table 1 shows the aggregate properties, test specifications, and standard limits. All aggregate properties were within standards set by RHD and BS. Stone chips were dried to a constant temperature of from 105 °C to 110 °C (220 °F to 230 °F) and separated by dry-sieving into the desired size fraction for the aggregate preparation.

### 2.4. Preparation of MWIFA and SD Fillers

**Figure 3:**
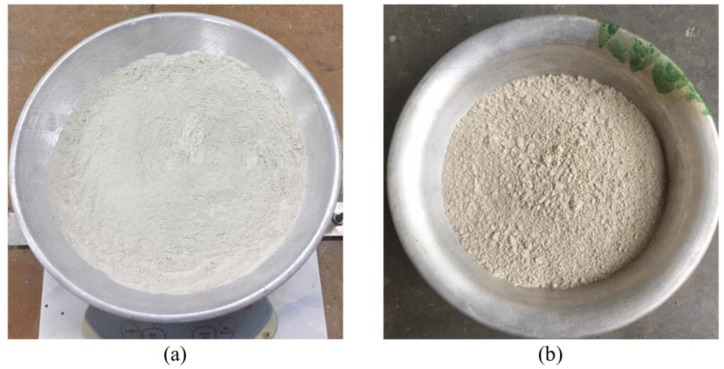
Images of (**a**) MWIFA and (**b**) SD filler materials.

The methodology proposed by Tang et al. [46] was implemented to process the fly ash sample into filler material. Fly ash samples were dried at 105 °C for 24 h, cooled at room temperature, and passed through ASTM standard test sieves (#4, #8, #16, #30, #50, #100, and #200) using a mechanical sieve shaker. As per ASTM D242 [47], finely separated fly ash or stone dust with a mass ranging from 70% to 100% passing through a #200 (75 μm) sieve can be used as mineral filler in asphalt mixes. This study used the sample portion passing through the #200 sieve (75 microns) in asphalt mixtures as a mineral filler. The SD filler was collected from the local market and similarly processed. Pictures of both fillers after sieving are shown in Figure 3a,b. The filler samples were stored in an air-tight container to keep them dry before experiments.

### 2.5. Properties of Mineral Fillers

Table 2 presents the chemical composition of MWIFA and SD obtained from XRF-Spectrometer analysis. It can be seen that the significant elements of MWIFA are CaO, SiO_2_ and SO_3_, while the significant elements of SD are SiO_2_, CaO, Al_2_O_3_, Fe_2_O_3_ and MgO. In MWIFA, the (SiO_2_ + Al_2_O_3_ + Fe_2_O_3_) content is 14.40%, less than 50%, and SiO_3_ exceeds 5%. According to ASTM C618-19 [48] standard classification, MWIFA cannot be considered class F or C fly ash. The chemical composition of mineral filler controls the filler properties and affects the adhesion properties of the asphalt mixtures [49]. As fly ash comprises a high content of CaO (62.39%), it can be used in asphalt mixtures with highly adhesive aggregates and a bituminous binder, positively affecting mixture stability [31]. The specific gravity of SD filler is 2.79, which is slightly higher than that of MWIFA (sp. gravity = 2.57). ASTM C188-16 [50] and ASTM D854-02 [51] standard test procedures were followed to determine the specific gravity of MWIFA and SD fillers, respectively.

**Figure 4:**
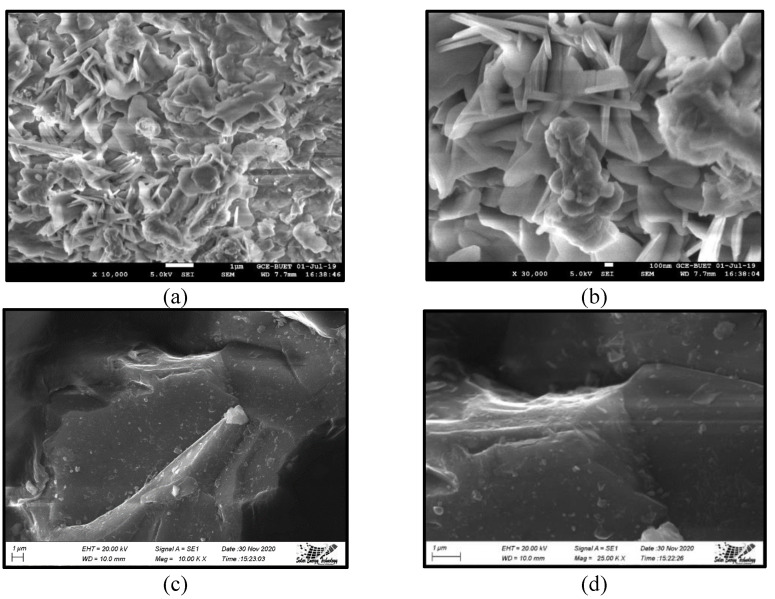
SEM images of (**a**) MWIFA filler (10,000× magnification) (**b**) MWIFA filler (30,000× magnification) (**c**) SD filler (10,000× magnification) (**d**) SD filler (25,000× magnification).

The external morphology (texture) and particle shape analyzed using SEM are shown in Figure 4a–d (Figure S1). The SEM images of MWIFA reveal that the particles have irregular shapes and assorted sizes. The surface texture of MWIFA seems rough, and the internal space between particles can be visibly detected. In contrast, the particles of SD have angular and prismatic shapes with smooth surface textures.

### 2.6. Marshall Mix Design

Three different types of specimens, namely (a) reference specimen using conventional filler (stone abrasion dust), (b) modified specimen using varying proportions of MWIFA as filler, and (c) control specimen without any filler, were prepared for testing as per ASTM D6926-20 [52] to observe and compare the effect of using MWIFA instead of conventional filler material. Filler contents were varied to determine the optimum filler content as 0% (control), 2%, 4%, 6%, 8%, and 10%, slightly extending the recommended filler range by Asphalt Institute. The specimens were prepared with 4.0%, 4.5%, 5.0%, 5.5%, and 6.0% of the binder for each proportion of filler. All specimens were tested according to ASTM D1559 [53] (Marshall Mix Design Method). The Marshall stability and flow tests were performed to determine the mechanical properties of the samples according to ASTM D6927-15 [54], and their corresponding maximum load resistance and flow values were recorded. The bulk specific gravity and density, percent air voids, and theoretical maximum specific gravity were determined for the volumetric analysis of each specimen. 

### 2.7. Immersion Test

Following the methodology proposed by Akbulut et al. [55], Marshall immersion tests were performed to inspect the deviations in the properties of hot bituminous mixtures under the effect of moisture. Specimens with varying filler ratios were produced using their optimum bitumen contents and cured for 48 h in a water bath at 60 °C. After the curing, the Marshall stability test was performed. The stability loss is defined as the reduction in stability after immersion in hot water for 48 h.

### 2.8. Determination of Optimum Filler Percentage 

If the filler ratio is not optimized in hot bituminous mixtures, it can adversely affect the performance of the mix [56]. The optimum filler content is determined using Equation (1) [55] as follows:

(1) $$\mathrm{Optimum}\;\mathrm{Filler}\;\mathrm{Content}\;(\%)\;=\frac{\left(F_{s}+F_{mi}+F_{d}+F_{v}\right)}{4}$$

Here, *F_s_* is the filler content corresponding to maximum stability; *F_mi_* is the filler content corresponding to minimum stability loss (determined from the Marshall mechanical immersion test); *F_d_* is the filler content corresponding to maximum unit weight; *F_v_* is the filler content corresponding to the minimum percentage of voids in mineral aggregate. *F_s_* is selected to obtain the maximum stability, *F_mi_* is selected to ensure minimum water susceptibility, and the other two parameters are selected to obtain the most tightly packed mix.

### 2.9. Leaching Test 

USEPA 1311 [57] protocol (Toxicity Characteristics Leaching Procedure (TCLP)) was used to determine the leaching potential. Samples were dried in an oven at 105 °C until constant weight, lightly ground for homogenization and crushed to a particle size smaller than 9.5 mm. The extraction fluid (pH of 2.88 ± 0.05) was added to a zero-headspace extractor (ZHE) at a liquid–solid ratio of 20:1, and the samples were agitated with a National Bureau of Standards (NBS) rotary tumbler for 18 h at 30 ± 2 rpm. The leachate was filtered with 0.45 μm pore size filter paper and analyzed for selected heavy metals (As, Cr, Cd, Cu, Hg, Ni, Pb and Zn) using Atomic Absorption Spectroscopy (AAS) (Shimadzu AA 6800). The Dutch tank test (NEN 7345 [58]) was used to evaluate the leaching performance of stabilized samples over a large period (64 days). Two leaching limits (U1 and U2) were used to categorize the environmental impact of the materials [59]. The sample was put in a polyethylene container and filled with acidified water (HNO_3_ at pH = 4). The leachate was removed and replaced with fresh extractant fluid eight times after 0.25, 1, 2.25, 4, 9, 16, 36, and 64 days. Leachate obtained from each extraction was analyzed for heavy metals. Equation (2) was used to compute the leachability of each pollutant (heavy metals) at the *i*th extraction [60].

(2) $$E_{i}=\frac{\left(C_{i}-C_{o}\right)V}{1000A}$$

Here, *E_i_* = leachability of a pollutant at the *i*-th extraction (mg/m^2^), *C_i_* = pollutant concentration at the *i*-th extraction (mg/L), *C_o_* = pollutant concentration in the blank (mg/L), *V* = volume of extractant agent (L), *A* = surface area of the sample (m^2^). 

After eight extractions, Equation (3) was used to compute the leachability (*E*) for the heavy metals [60].

(3) $$E=\sum_{i=1}^{8}E_{i}$$

## 3. Results and Discussion

### 3.1. Unit Weight

**Figure 5:**
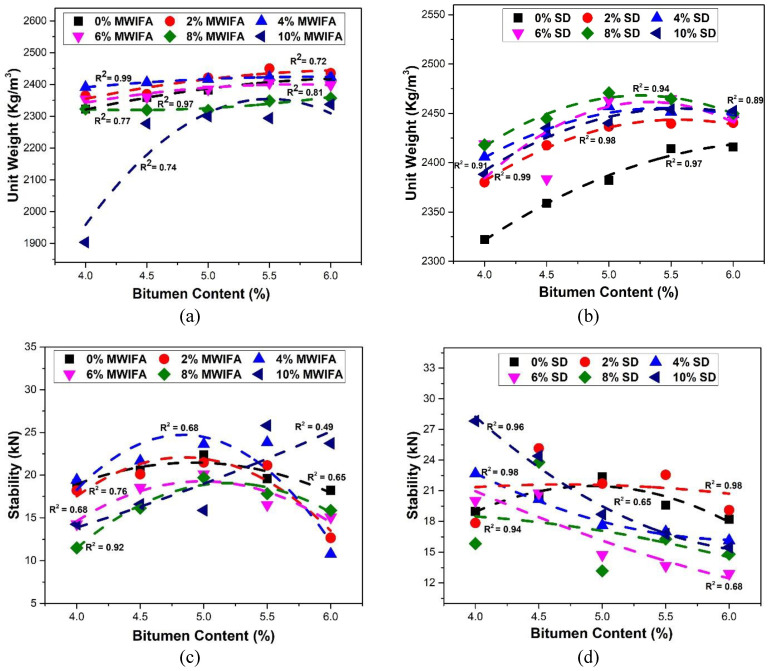
Relationships between unit weight and bitumen content for (**a**) MWIFA filler and (**b**) SD filler, and between Marshall stability and bitumen content for (**c**) MWIFA filler and (**d**) SD filler.

The relationship between unit weight and the bitumen content in the bituminous mixes for MWIFA and SD filler is shown in Figure 5a,b. The unit weight increased with the increase in asphalt content for both fillers. The increasing bitumen content fills the voids, increasing the unit weight in the mix [15]. Similar results were observed in the studies using fly ash, SD, brick dust and cement as fillers in the hot bituminous mixes [15,61,62]. In the case of MWIFA, the maximum unit weight was found in 4% filler (8% for SD filler), indicating that the most compact mix is obtained in this filler ratio. MWIFA enters the voids between sand particles, thus raising the density and unit weight. However, MWIFA, being more irregularly shaped than SD, thrusts out the sand particles while forming more voids, consequently decreasing the unit weight. Mazumdar and Rao [63] observed similar behavior with other fly ash forms. 

### 3.2. Stability

The stability property of the bituminous mix indicates the pavements’ resistance to traffic-induced stresses [55]. The relationships between the stability values and bitumen contents for MWIFA and SD fillers are depicted in Figure 5c,d. The stability values of all hot mix samples, except the one with 10% MWIFA, initially increase with bitumen content and decrease after reaching a peak. The 2% and 8% SD samples follow the same pattern. Sutradhar et al. [14], Kar et al. [61], Saltan et al. [11], Jony et al. [64], Rahman et al. [62], and Mistry and Roy [22] found similar stability results for their respective experiments with asphalt mixes. On the other hand, the stability values decrease with increasing asphalt binder content for 4%, 6%, and 10% SD filler ratios. Although the stability graphs of MWIFA and SD fillers follow different trends, all the Marshall stability values meet the minimum Marshall mix design criteria (5.34 kN) recommended by the Asphalt Institute.

The maximum stability values of mixes with 0%, 2%, 4%, 6%, 8%, and 10% MWIFA filler are found to be 22.37 kN, 21.47 kN, 23.82 kN, 20.11 kN, 19.70 kN, and 25.80 kN, respectively. Fly ash filler goes into the voids of FA and interlocks the particles, which may cause an initial increase in stability values [63]. The maximum stability values of mixes with 2%, 4%, 6%, 8%, and 10% SD filler are 25.15 kN, 22.68 kN, 20.75 kN, 23.78 kN, and 27.82 kN, respectively. The bitumen content corresponding to the maximum stability is higher for the mixes containing MWIFA filler than those with SD filler, and the maximum stability values of SD filler mixes are comparatively higher, as seen in Figure 5c,d. For example, if we choose a 2% filler content, the corresponding bitumen content for maximum stability of the MWIFA mix (21.47 kN) is 5%, while for maximum stability of the SD mix (25.15 kN), it is 4.5%. This phenomenon is the same for other filler contents. This may be because SD filler produces a viscous asphalt cement mixture with lower bitumen content [15]. It is possible that the greater dispersion of binders in asphalt mixes having SD as a filler confers more stiffness and, consequently, more stability [65].

### 3.3. Flow

**Figure 6:**
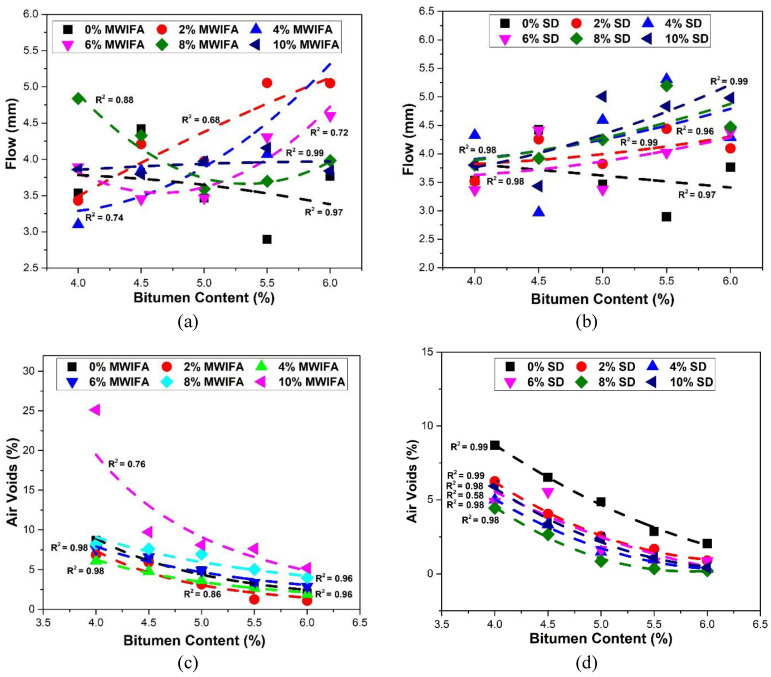
Relationships between Marshall flow value and bitumen content for (**a**) MWIFA filler and (**b**) SD filler, and between air voids and bitumen content for (**c**) MWIFA filler and (**d**) SD filler.

The flow value denotes the vertical deformation under maximum load. It signifies that bituminous mixtures’ plasticity and flexibility properties are inversely related to internal friction [11]. Figure 6a,b illustrates the relationship between the Marshall flow value and bitumen content with varying MWIFA and SD fillers. The flow values for both filler materials, except the 8% MWIFA filler ratio, follow the general trend of a consistent rise with the increasing bitumen contents. Uzun and Terzi [66], Sutradhar et al. [15] and Kar et al. [61] found that the flow values increased with the increase in bitumen contents in their studies. For the case of 8% MWIFA, the decrease in flow values may be ascribed to the increased interlocking offered by fly ash particles, and the successive rise in the flow values may be because of the large surface area, resulting in insufficient coating [63]. All the flow values for all filler percentages closely comply with the Marshall mix design limit (from 2 mm to 4 mm) of the Asphalt Institute [67].

### 3.4. Air Voids

The presence of air voids in a dense-graded mix prevents the pavement from flushing, shoving, and rutting. Figure 6c,d shows the relationship between the percentage of air void and bitumen content with MWIFA and SD fillers. The percentage of air voids decreases with the increase in bitumen contents for both fillers. An increased bitumen content reduces air voids by filling more voids in the paving mixture. Nayak and Mohanty [68], Uzun and Terzi [66], Kar et al. [61], and Mazumdar and Rao [63] found a similar decreasing trend of air voids with increased bitumen content, with fly ash and SD as mineral fillers. Adding filler to hot bituminous mixtures eases the compensation of fine aggregates in the mix, and thus voids in the mixtures reduce with the increase in filler proportions [15]. Except for a few ratios, mixes with MWIFA filler ratios have comparatively higher air voids values than those with the same SD filler ratios. The differences in size, shape, surface structure and physio-chemical properties between the MWIFA and SD fillers could be responsible for this Marshall property variation [62,69]. Zulkati et al. [24] mentioned that some fillers create stiff asphalt mastic and require greater compaction effort. It is possible that SD, being less fine than MWIFA, has lower air void values in Marshall samples despite having the same mix proportions and compaction energies. All the air voids values except for a few percentages for both fillers are within the standard Marshall mix design limit (3 to 5), and an OBC value was calculated from the test results for each filler type and ratio according to the Marshall mix design method.

### 3.5. Voids in Mineral Aggregate (VMA)

**Figure 7:**
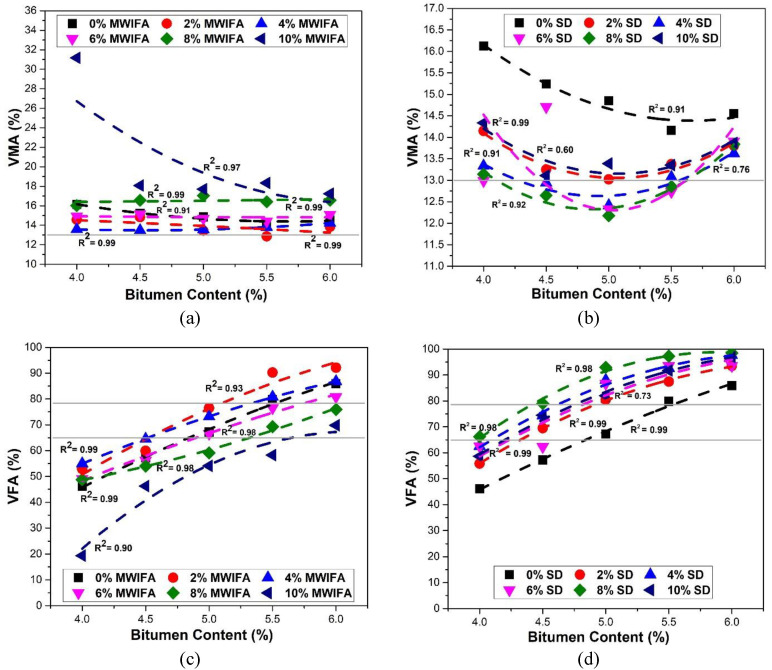
Relationships between voids in mineral aggregate and bitumen content for (**a**) MWIFA filler and (**b**) SD filler, and between voids filled with asphalt and asphalt content for (**c**) MWIFA filler and (**d**) SD filler. The horizontal line in (**a**,**b**) represents the minimum Marshall mix design requirement for VMA. The horizontal lines in (**c**,**d**) represent the upper and lower limits of VFA for the Marshall mix design.

An adequate VMA is necessary to ensure the film thickness within the mix without too much asphalt bleeding or flushing, ensuring durability in the mix [66,70]. Figure 7a,b depicts the relationship between VMA (%) and bitumen content with varying MWIFA and SD fillers. All the VMA (%) values for both fillers, except a few values for SD filler, satisfy the Marshall minimum design requirement of 13% (the horizontal line in Figure 7a,b) for VMA recommended by the Asphalt Institute. The VMA has been found to decrease with increasing asphalt content, reach a minimum, and subsequently increase for both fillers except for the 6% and 8% of MWIFA filler ratios. VMA initially decreases due to better compaction and rises again as the extra bitumen in the mix pushes apart the aggregates [67]. Previous studies found a decreasing trend of %VMA values to the increasing bitumen contents in paving mixtures with various fly ashes and SD as mineral fillers [15,61,62,64].

### 3.6. Voids Filled with Asphalt (VFA)

The VFA property regulates the plasticity, durability, and friction coefficient of the bituminous mixtures. The relationships between VFA and bitumen contents for MWIFA and SD fillers percentages are shown in Figure 7c,d. The %VFA values of compacted mixtures increase with bitumen contents for both fillers. This trend is consistent with previous studies of paving mixtures with various fly ashes and SD as mineral fillers [12,15,61,64,66]. The VFA values for all samples are not within the Marshall mix design criteria of 65–78% (horizontal lines in Figure 7c,d), specified by the Asphalt Institute. However, the VFA design value obtained for the corresponding filler ratio is within the standard limit.

### 3.7. Marshall Properties at Optimum Bitumen Content (OBC)

The OBC for each filler percentage is defined as the respective bitumen content at 4% air voids. The properties of the mixes at their OBC with each filler type and contents are shown in Table 3. All the OBC levels satisfy the Roads and Highway Department, Bangladesh standard limit. No particular trend was observed in OBC values with the increase in MWIFA or SD fillers (Table 3). It appears that, apart from 2% and 4% MWIFA fillers, there is an increasing trend for OBC, but no such trend was observed for SD fillers. The determination of OBC employs a graphical method (corresponds to 4% air voids in the graph). If there are limited data, the determination could have some anomalies. It is possible that such anomalies masked the effect of the varying filler ratios. However, the OBC requirement of MWIFA filler mixes was consistently higher than that of SD filler. Joumblat et al. [34] also found a slight increase in the OBC values for the samples modified with municipal solid waste incineration fly ash. Fly ash absorbs slightly more bitumen than SD; therefore, it needs more asphalt to bind [61]. The high porosity, specific surface area, surface roughness, and particle shape of the incineration fly ash can cause this phenomenon [34].

The Marshall Stability values of the mixes with MWIFA filler at the optimum bitumen content are 15 kN to 23 kN (Table 3). On the other hand, the stability values vary from 17 kN to 25.5 kN for SD filler. Therefore, the SD filler exhibits slightly higher stability than the MWIFA filler at OBC for most filler contents. However, the stability values of both fillers at OBC meet the minimum Marshall mix design requirement of 5.34 kN.

In mixes including MWIFA filler, Marshall flow values at their respective OBC are from 3.22 mm to 4.00 mm, whereas, for SD filler, this range is within 3.51–4.24 mm (Table 3). The flow values of hot bituminous mixtures used in medium traffic surface and base must be between 2 mm and 4 mm according to the Marshall mix design criteria of the Asphalt Institute. The flow values of the two fillers at OBC generally conform to the Marshall mix design limit. VFA for the OBC with 0%, 2%, 4%, 6%, 8% and 10% MWIFA filler is within 70.45–75.83%. For SD filler samples, this range is from 69.37% to 71.95% (Table 3). The VFA values for both fillers at OBC have satisfied the Marshall mix design maximum and minimum VFA requirements. In mixes including 0%, 2%, 4%, 6%, 8% and 10% MWIFA filler, the VMA corresponding to the optimum level of bitumen is found within 13.54–16.70% (Table 3). Design %VMA increases with an increase in MWIFA filler content. A similar trend in VMA at OBC is also observed by Jony et al. [64] and Sargın et al. [71] in their respective studies. For SD filler samples, this range is from 13.02% to 13.77% (Table 3). The VMA values for samples with MWIFA are slightly higher than those with SD as filler. Joumblat et al. [34] observed a similar result, where all samples modified with municipal waste incineration fly ash showed higher VMA values. The VMA values for both fillers at OBCs complied with the Marshall mix design minimum requirement of 13%.

### 3.8. Marshall Immersion

Mechanical immersion tests determine the loss of stability in hot bituminous mixtures under moisture action. At OBC, there is no definite trend with stability loss in immersion with the increase in filler content. However, mixes with MWIFA showed less immersion loss than SD (Table 3). The Marshall stability loss is the lowest for the mix containing 8% MWIFA among all mixtures prepared with both fillers (Table 3). Carpenter [72] found that fly ash favored retaining the compressive strength of asphalt concrete immersed in water. The likely reason for this is that the predominant constituent in MWIFA is CaO, which exhibits water-resistive properties regarding moisture stability in bituminous mixes [73]. On the other hand, the asphalt mixture prepared with SD had comparatively low moisture resistance and poor adherence with asphalt binder because of its high presence of SiO_2_ [49,74]. Akbulut et al. [55] found a similar trend of stability losses with the increasing granite sludge filler ratios and obtained the minimum stability loss in the 8% filler-containing specimens.

### 3.9. Optimum Filler Content

The Optimum filler content (OFC), calculated using Equation (1), which corresponds to maximum stability, lowest Marshall stability loss, maximum density and the lowest percentage of voids in the hot bituminous mixtures, is 5.5% and 9%, respectively, for MWIFA and SD fillers. The required optimum filler amount is lower in the asphalt mixes with MWIFA filler than those containing SD filler. Several studies using fly ash as a mineral filler obtained OFC values between 4% and 7% and exhibited better performance than conventional fillers [67,75,76].

### 3.10. Heavy Metal Leaching

The concentration of leachates from raw fly ash and Marshall samples in standard TCLP leaching test and a comparison with Land Disposal Restrictions Limits (LDR) for hazardous wastes are given in Table 4. The concentrations of the heavy metals found in raw fly ash and asphalt samples with MWIFA filler are far below the USEPA regulatory limits. The maximum amount of As, Cr and Zn metals in MWIFA that could be reduced was 37.3%, 94.4%, and 100%, respectively, when using a 2% filler in a bituminous mix. The highest reduction for Pb (57.7%) was found in the mix containing 4% MWIFA filler, while the maximum reduction in Cd (69.8%) was observed in the 6% MWIFA filler sample. There was an increase in copper and nickel metals, probably from other constituents, but it did not exceed the EPA Land Disposal limit. These results ensure that the leaching tendency of the heavy metals from the asphalt paving mixture incorporating MWIFA is significantly lower than in raw MWIFA. This suggests that MWIFA can be reliably used in paving mixtures without any concerns for environmental hazards.

The cumulative leached concentrations of all the heavy metals were determined using the Dutch tank test and summarized in Table 5. According to NEN 7345, if the cumulative heavy metal concentrations of stabilized samples are below U1, the stabilized waste can be used on land and construction material without restriction [59,77]. All the cumulative concentrations are found far below the regulatory limit U1. Heavy metals (Cd, Ni, Zn, Cu and Pb) leached insignificantly from monolithic asphalt specimens in acidic water. Therefore, the inclusion of MWIFA in asphalt pavement can be considered environmentally friendly.

## 4. Conclusions

### 4.1. Mechanical and Sustainable Performance

The study evaluates the environmental and physical performances of the bituminous mixes prepared with MWIFA as mineral filler. The study concludes with the following findings:

(1) All OBC values for mixes with MWIFA fall within the specified limits of the Roads and Highways Department, Bangladesh, depicting compliance with the existing practices. The Marshall properties, such as stability, flow, air voids, VMA and VFA at respective OBCs, satisfy the criteria recommended by the Asphalt Institute for each of the varying MWIFA filler ratios. MWIFA performs similarly to SD, verifying its potential as an alternative filler in bituminous courses, especially in a country where the source of traditional filler is limited.

(2) The OFC values for MWIFA and SD fillers are 5.5% and 9%, respectively. The bituminous mixes with a 5.5% MWIFA filler would perform better in pavements, whereas those with a 9% SD filler will exhibit the same performance. The optimum filler required in asphalt concrete mixes for MWIFA is less than that of SD filler. So, the MWIFA filler could be a promising substitute for SD, especially where SD is imported with foreign currency.

(3) The Marshall stability loss of mixes with MWIFA is less than that of SD, showing its ability to protect against the moisture effect. So, using MWIFA as a mineral filler in the pavement can be more suitable than conventional SD filler, especially in tropical areas.

(4) Leaching test results depict no environmental restrictions on using MWIFA in asphalt pavement as filler. Long-term heavy metal leaching is negligible. The incorporated MWIFA–asphalt matrix reduces the leachability of the toxic heavy metals contained in the MWIFA. MWIFA will have no adverse impact on the environment after stabilization.

### 4.2. Practical Implications on the Utilization of MWIFA

From the evaluation of the test results, MWIFA can be used efficiently as a mineral filler in the asphalt paving mix as a replacement for conventional SD filler, especially in areas where MWIFA is abundantly available with affordable transportation costs. This can also be an eco-friendly solution to medical waste disposal problems, especially for a country with a scarcity of land to provide a landfill area. However, effective guidelines and policies from the local government are needed to avoid potential confusion regarding its use. Such measures would lead to the greater consumption of MWIFA in the pavement industry and reduce the demand for virgin materials, resulting in sustainable waste management.

### 4.3. Limitations and the Scope for the Future Studies

The conclusion of the paper is based on findings from environmental tests as well as the observation of Marshall properties. Future works should consider some of the mechanical properties obtained from Indirect Tensile Strength (ITS), Indirect Tensile Stiffness Modulus (ITSM), Retained Marshall Stability (RMS), and Dynamic Modulus tests to assess the long-term impact of MWIFA’s incorporation in asphalt mixes.

## Figures and Tables

**Table 1:** Properties of bitumen and aggregate.

Properties	Test Designation	Sample Values	Standard Specifications
Bitumen			
Penetration at 25 °C (0.1 mm)	AASHTO T49	61	60–70 ^a^
Flash Point (°C)	AASHTO T48	295	min 232 ^a^
Ductility at 25 °C (cm)	AASHTO T51	100+	min 100 ^a^
Solubility in Trichloroethylene (%)	AASHTO T44	97.8	min 99.0 ^a^
Loss on Heating (%)	AASHTO T179	0.06	<0.8 ^a^
Softening Point (°C)	AASHTO T53	49	48–56 ^a^
Aggregate			
Aggregate Impact Value (%)	BS 812-3	28	<30 ^b^
Aggregate Crushing Value (%)	BS 812-3	17	<30 ^c^
Ten Percent Fine Value (KN)	BS 812-111	130	min 100 ^b^
Flakiness Index (%)	BS 812-105.1	26	<30 ^b^
Elongation Index (%)	BS 812-105.2	29	<30 ^b^
Angularity Number	BS 812-1	11	0–12 ^b^
Los Angeles Abrasion (%)	AASHTO T96	31	<35 ^c^
Specific Gravity (CA)	AASHTO T85	2.72	-
Specific Gravity (FA)	AASHTO T84	2.6	-

^a^ AASHTO M 20-70 (2004). Standard Specification for Penetration Graded Asphalt Cement. American Association of State and Highway Transportation Officials. ^b^ BS 882 (1992). Specification for aggregates from natural sources for concrete. ^c^ RHD (2011). Standard specifications for pavement work, Ministry of Communications Roads and Highways Department, Bangladesh.

**Table 2:** Chemical composition (wt%) of MWIFA and SD.

Chemical Components	MWIFA	SD
CaO	62.39	25.53
SiO_2_	8.92	51.71
SO_3_	5.92	0.61
Na_2_O	5.35	0.10
TiO_2_	3.73	0.79
Al_2_O_3_	3.73	6.17
MgO	2.65	5.50
ZnO	2.13	-
Fe_2_O_3_	1.75	6.11
P_2_O_5_	1.38	0.19
K_2_O	1.19	2.16
NiO	0.50	-
Cr_2_O_3_	0.21	0.07
MnO	0.07	0.10
CuO	0.04	-
Br	0.03	-
ZrO_2_	-	0.01
SrO	-	0.05

**Table 3:** Volumetric and Marshall properties of bituminous mixes at OBC content.

Design Criteria	OBC (%)	%V_a_	%VMA	%VFA	Stability (kN)	Flow (mm)	Stability Loss (%)
0% Filler	5.22	4	14.55	72.66	21.16	3.22	32.91%
2% MWIFA Filler	4.85	4	13.86	71.49	21.06	4.00	7.59%
4% MWIFA Filler	4.84	4	13.54	70.45	22.99	3.92	35.12%
6% MWIFA Filler	5.3	4	14.53	72.52	17.98	3.96	24.9%
8% MWIFA Filler	5.99	4	16.55	75.83	15.88	3.98	0.70%
10% MWIFA Filler	6.25	4	16.70	75.48	22.69	3.69	23.56%
2% SD Filler	4.51	4	13.24	69.80	25.06	4.24	31.35%
4% SD Filler	4.3	4	13.10	69.55	21.18	3.51	30.86%
6% SD Filler	4.7	4	13.77	71.95	18.37	4.00	21.86%
8% SD Filler	4.12	4	13.02	69.37	17.79	3.83	29.18%
10% SD Filler	4.37	4	13.42	70.47	25.27	3.53	12.52%
Standard limit ^a b^	4.90–6.5 ^a^	3–5 ^b^	min 13 ^b^	65–78 ^b^	min 5.338 ^b^	2–4 ^b^	-

^a^ RHD (2011). Standard specifications for pavement work, Government of the People’s Republic of Bangladesh Ministry of Communications Roads and Highways Department, Bangladesh. ^b^ Asphalt Institute (2014). MS-2 asphalt mix design methods (7th Edition). Asphalt Institute.

**Table 4:** TCLP test results of raw MWIFA and MWIFA filler (units are in ppm, except heavy metals reduction (%)).

Heavy Metals		As	Pb	Cu	Cr	Cd	Zn	Ni	Hg
Raw MWIFA		0.0298	0.169	0.003	0.054	0.106	0.011	0.003	ND ^a^
MWIFA as filler in Marshall Samples	2% filler	0.0187	0.16	0.059	0.003	0.089	0	0.377	ND
	Heavy Metal Reduction (%)	37.3	5.3	-	94.4	16.0	100	-	-
	4% filler	0.0254	0.072	0.025	0.067	0.042	0.001	0.087	ND
	Heavy Metal Reduction (%)	14.8	57.4	-	-	60.4	90.9	-	-
	6% filler	0.0256	0.08	0.019	0.027	0.032	0.002	0.076	ND
	Heavy Metal Reduction (%)	14.1	52.7	-	50	69.8	81.8	-	-
	8% filler	0.0688	0	0.01	0.009	0.085	0	0.045	ND
	Heavy Metal Reduction (%)	-	100	-	83.3	19.8	100	-	-
	10% filler	0.0251	0.125	0.012	0.07	0.048	0.002	0.15	ND
	Heavy Metal Reduction (%)	15.8	26.0	-	-	54.7	81.8	-	-
EPA Land Disposal Restriction for Hazardous Waste ^b^	Universal Treatment Standards limit	5	0.75	-	0.6	0.11	4.3	11	0.2
	Toxicity Characteristic Regulatory Limit	5	5	-	5	1	-	-	0.2

^a^ ND: Not Detected. ^b^ USEPA (1996). Land Disposal Restrictions for Hazardous Waste, United States Environmental Protection Agency.

**Table 5:** Results of the tank leaching tests in Marshall samples after eight extractions.

Heavy Metals	Cd	Ni	Zn	Cu	Pb
Unit	mg/m^2^	mg/m^2^	mg/m^2^	mg/m^2^	mg/m^2^
2% MWIFA	0.00018	0.00028	0.00002	0.00005	0.00034
4% MWIFA	0.00034	0.00022	0.00002	0.00005	0.00018
6% MWIFA	0.00022	0.00028	0.00001	0.00008	0.00040
8% MWIFA	0.00035	0.00027	0.00003	0.00015	0.00022
10% MWIFA	0.00022	0.00033	0.00002	0.00006	0.00041
Leaching limits as per NEN 7345 [58]					
U1	1	50	200	50	100
U2	7	350	1500	350	800

## Data Availability

All data generated or analyzed during the study are included in this manuscript.

## Supplementary Materials

The following supporting information can be downloaded at: https://www.mdpi.com/article/10.3390/ma16165612/s1, Figure S1: SEM image of MWIFA with 500× zoom; Figure S2: Prepared samples: (a) after demolding and (b) during submerged for SSD weight calculation. Table S1: Mix proportions of the asphalt mixes for both fillers.
