# Supplementary material for: Medical Waste Incineration Fly Ash as a Mineral Filler in Dense Bituminous Course in Flexible Pavements
Source: Materials (Basel). 2023 Aug 13;16(16):5612. doi: 10.3390/ma16165612 (PMC10456925; doi:10.3390/ma16165612)
Supplement: Supplementary file 1 [file materials-16-05612-s001.zip › materials-2516848-supplementary.pdf]

Supplementary Information

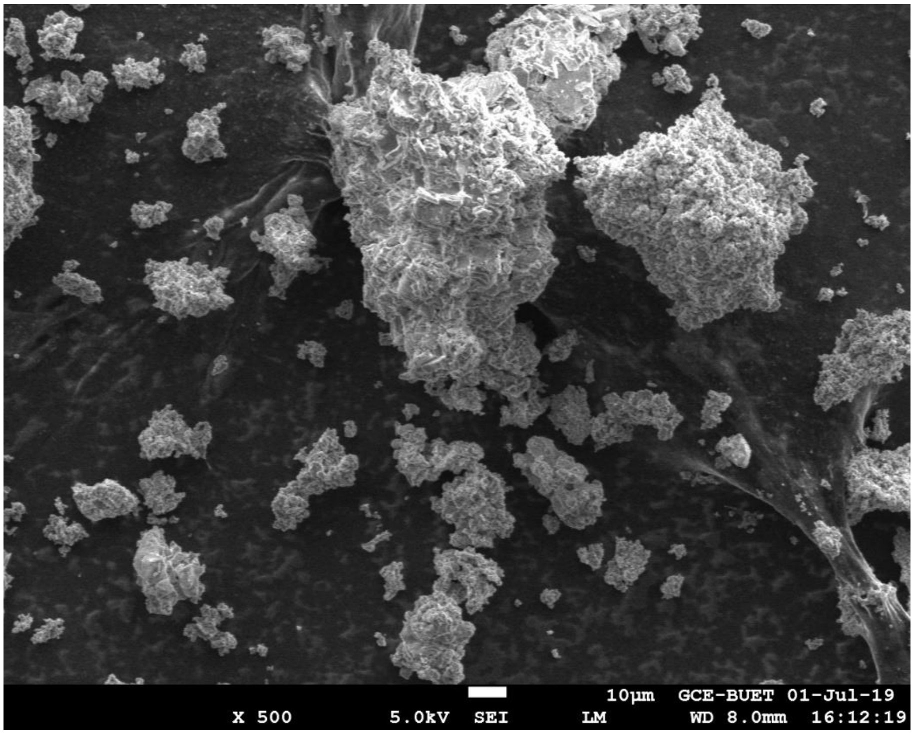

Figure S-1: SEM image of MWIFA with 500x zoom.

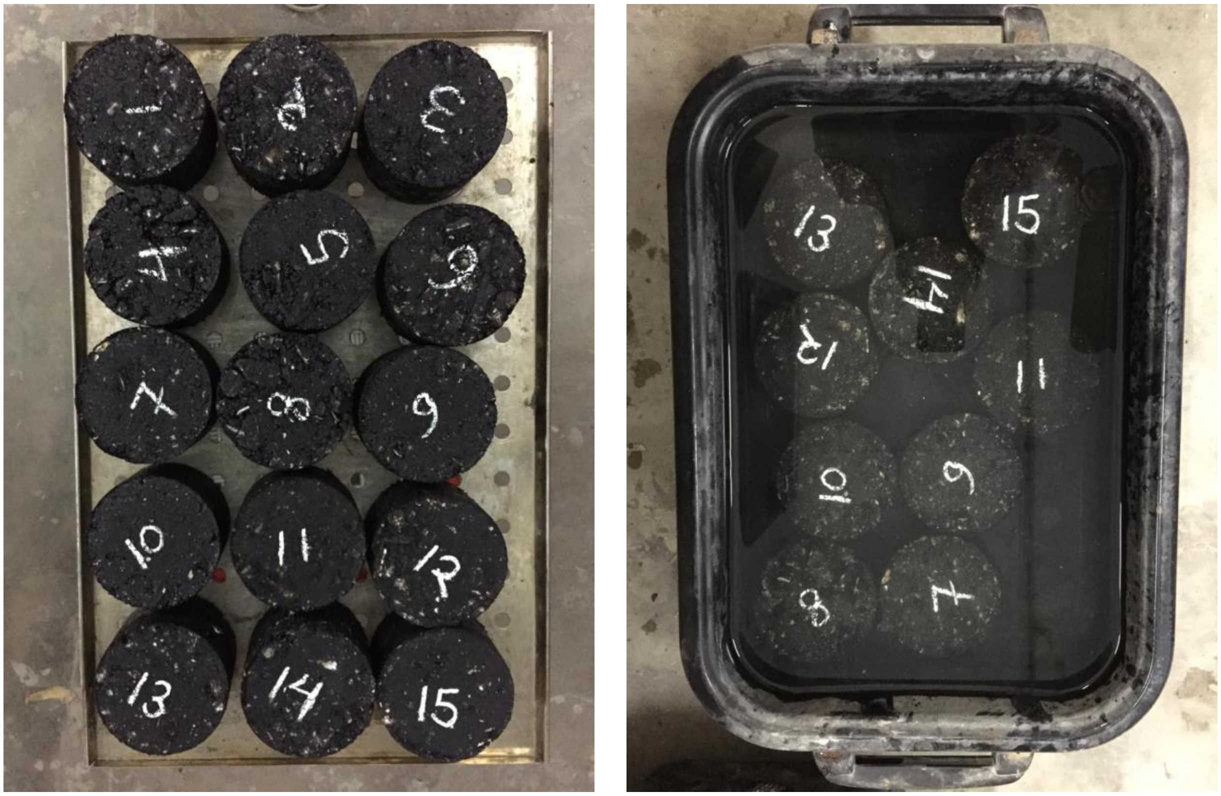

(a) (b)

Figure S-2: Prepared samples: (a) after demolding and (b) during submerged for SSD weight calculation.

Table S-1: Mix proportions of the asphalt mixes for both fillers.

Sieve Size	0% Filler	2% Filler	4% Filler	6% Filler	8% Filler	10% Filler
	% Passing	% Passing	% Passing	% Passing	% Passing	% Passing
1 inch (25 mm)	100	100	100	100	100	100
3/4 inch (19 mm)	95	95	95	95	95	95
3/8 inch (9.5 mm)	68	68	68	68	68	68
No. 4 (4.75 mm)	50	50	50	50	50	50
No. 8 (2.36 mm)	35	35	36	36	37	38
No. 50 (0.3 mm)	9	10	12	13	15	17
N0. 200 (0.075 mm)	0	2	4	6	8	10
